# Occurrence of the leucine-to-phenylalanine knockdown resistance (*kdr*) mutation in *Anopheles arabiensis *populations in Tanzania, detected by a simplified high-throughput SSOP-ELISA method

**DOI:** 10.1186/1475-2875-5-56

**Published:** 2006-07-05

**Authors:** Manisha A Kulkarni, Mark Rowland, Michael Alifrangis, Frank W Mosha, Johnson Matowo, Robert Malima, Justin Peter, Eliningaya Kweka, Issa Lyimo, Stephen Magesa, Ali Salanti, Manfred E Rau, Chris Drakeley

**Affiliations:** 1 Department of Natural Resource Sciences, McGill University, Macdonald Campus, Ste Anne de Bellevue, Quebec H9X 3V9, Canada; 2 Joint Malaria Programme, P.O. Box 2228, Moshi, Tanzania; 3 National Institute for Medical Research, Amani Research Centre, Tanzania; 4 Kilimanjaro Christian Medical Centre, P.O. Box 3010, Moshi, Tanzania; 5 Centre for Medical Parasitology at Institute of Medical Microbiology and Immunology and Institute of Public Health, University of Copenhagen, Denmark; 6 London School of Hygiene and Tropical Medicine, Keppel Street, London WC1E 7HT, UK

## Abstract

**Background:**

Molecular markers of insecticide resistance can provide sensitive indicators of resistance development in malaria vector populations. Monitoring of insecticide resistance in vector populations is an important component of current malaria control programmes. Knockdown resistance (*kdr*) confers resistance to the pyrethroid class of insecticides with cross-resistance to DDT through single nucleotide polymorphisms (SNPs) in the voltage-gated sodium channel gene.

**Methods:**

To enable detection of *kdr *mutations at low frequency a method was developed that uses polymerase chain reaction (PCR) and enzyme-linked immunosorbent assay (ELISA)-based technology, allowing rapid, reliable and cost-effective testing of large numbers of individual mosquitoes. This was used to assay mosquitoes from sites in lower Moshi, Tanzania.

**Results:**

Sequence-specific oligonucleotide probes (SSOP) were used for simultaneous detection of both East and West African *kdr *mutations with high specificity and sensitivity. Application of the SSOP-ELISA method to 1,620 field-collected *Anopheles arabiensis *from Tanzania identified the West African leucine-phenylalanine *kdr *mutation in two heterozygous individuals, indicating the potential for resistance development that requires close monitoring.

**Conclusion:**

The presence of the West African *kdr *mutation at low frequency in this East African population of *An. arabiensis *has implications for the spread of the *kdr *gene across the African continent.

## Introduction

With efforts to scale-up the coverage of insecticide-treated nets (ITN) in Africa [1] there is increasing concern regarding the potential impact of insecticide resistance on malaria control [2,3]. The knockdown resistance (kdr) mechanism confers resistance to pyrethroid insecticides and DDT through point mutations in the voltage-gated sodium channel gene [4,5]. Resulting single amino acid changes in the domain II region of the sodium channel reduce the sensitivity of the insect nervous system to these compounds. Two different *kdr *mutations have been identified in resistant *Anopheles gambiae *s.l. populations from East and West Africa. The West African *kdr *is characterized by a leucine to phenylalanine substitution at position 104 of the voltage-gated sodium channel sequence (L104F) [4], while the East African *kdr *involves a serine substitution at the same position (L104S) [6]. Recent reports of both *kdr *mutations in *An. gambiae *from Uganda [7]indicate, however, that this geographic distinction may be too simplified. While the *kdr *allele has predominantly been found in *An. gambiae *s.s. [8-10], several reports have also identified the presence of *kdr *in the sibling species *Anopheles arabiensis *[7,11,12].

Models of antimalarial drug resistance spread have demonstrated the potential for a rapid increase in the frequency of resistance, rising quickly from undetectable levels to levels that result in control failure [13]. Models of insecticide resistance show an equally rapid rise when the frequency reaches levels as low as 0.1% [14]. Since the *kdr *allele is incompletely recessive, conventional bioassay methods that measure phenotypic resistance cannot reliably detect the heterozygous proportion of the population [8]. A more sensitive approach is direct genotyping, which can identify heterozygotes and, thus, facilitate early detection of resistance development.

However, current methods for *kdr *genotyping have limitations for testing large numbers of individuals, which is often needed in routine monitoring programmes. Multiplex polymerase chain reaction (PCR) methods [4,6] are time consuming and the visualization of the PCR product by gel electrophoresis uses toxic reagents. The PCR-dot blot method [15] is also relatively time consuming, and scoring of the product is subjective if done by eye, or requires costly equipment for automated scoring. The HOLA technique [16] allows more simple and reliable detection of *kdr *mutations, but requires two thermal cycling steps, necessitating additional time and cost. The FRET/MCA technique requires costly real-time PCR equipment [7], which is not affordable by most resource poor laboratories.

A rapid, high-throughput method for *kdr *screening was developed that is appropriate for use in laboratories in malaria endemic countries. This method combines a PCR assay with visualization of the product using sequence-specific oligonucleotide probes (SSOP) in an enzyme-linked immunosorbent assay (ELISA) format. It is based on the SSOP-ELISA method of Alifrangis et al. [17] for detecting drug resistance mutations in *Plasmodium falciparum*, with some modifications. The is rapid, allowing analysis of more than 150 samples in a single day, convenient, using a 96-well plate format for easy transfer of reagents and samples with multichannel pipettes, and cost-effective, amounting to roughly 1 USD per sample. Following optimization, the method was evaluated by analysis of field-collected material from northern Tanzania, where insecticide resistance monitoring and ITN evaluation activities are ongoing.

## Methods

### *Anopheles gambiae *reference strains

Three laboratory reference strains of *An. gambiae *s.s. were used to verify the specificity and sensitivity of the assay, and these were used as controls in subsequent experiments. R70 from Tanzania possesses the wildtype susceptible (104L) genotype; VKPR, homozygous for the L104F West African *kdr *allele; and RSP, homozygous for the L104S East African *kdr *allele. In addition, artificial heterozygotes were created by combining DNA extracted from individuals of susceptible and resistant strains, L(R70)/F(VKPR) for the West African *kdr *and L(R70)/S(RSP) for the East African *kdr*.

### Field-collected samples

Field studies were conducted in villages in the lower Moshi area on the slopes beneath Mt Kilimanjaro in northeast Tanzania.

#### Mabogini

Female *An. arabiensis *were collected by indoor-resting catch from houses in Mabogini village (3°22' S, 37°19' E) during June/July 2004, and exposed to 0.75% permethrin in WHO susceptibility tests [18]. Survivors and dead individuals from these tests (n = 822) were processed for circumsporozoite protein (CSP) ELISA for detection of *P. falciparum *sporozoites, i.e. head and thorax of individual mosquitoes homogenized in grinding buffer [19], then stored in 96-well plates in grinding buffer at -20°C. Experimental hut trials conducted in the same area to evaluate permethrin-treated nets and sheets provided survivors and dead mosquitoes (n = 156), which were stored on silica gel at 4°C.

#### Msitu wa Tembo

Female *An. arabiensis *(n = 642) from light trap collections carried out in 2004 in the village of Msitu wa Tembo, Tanzania (3°33' S, 37°17' E) were processed for CSP ELISA and stored in 96-well plates at -20°C.

### Extraction of DNA from mosquitoes

Individual mosquitoes, dried on silica gel or processed for CSP ELISA, were homogenized in 50 μl STE buffer (1 mM EDTA, 10 mM Tris-HCl, 50 mM NaCl), incubated at 95°C for 12 minutes then centrifuged at 13,000 rpm for four minutes at room temperature. Aliquots of the supernatant containing suspended DNA were transferred into fresh tubes and stored at -20°C until use. DNA extraction was carried out in individual tubes or in 96-well PCR plates using a clean pipette tip for homogenization of each sample. DNA extracted from individual, dried mosquitoes using the method described by Collins et al. [20] was used for comparison. Ten microlitres of the DNA extract from two individuals were pooled in each well, and this pooled DNA was used in the polymerase chain reaction.

### Polymerase chain reaction

Forward and reverse primers developed by Kolaczinski et al. [21] were used to amplify a 216 bp fragment of the voltage-gated sodium channel gene, with a biotin modification of the reverse primer at the 5' end (MWG Biotech, Riskov, Denmark). Each 20 μL PCR reaction consisted of 0.25 mM each dNTP, 0.1 μM each primer, one unit HotStarTaq polymerase (Qiagen, Albertslund, Denmark) in buffer containing 1.5 mM MgCl_2 _(Qiagen) and 2 μL extracted DNA. Reaction conditions were 94°C for 15 minutes followed by 35 cycles of 94°C for one minute, 55°C for one minute and 72°C for one minute, with a final extension at 72°C for four minutes; the PCR product was kept at 4°C until use. Amplifications were performed in 96-well PCR plates and the reaction mixture was overlaid with one drop of mineral oil. PCR products from controls and several samples from each plate were confirmed by electrophoresis on a 1.5% agarose gel. Species identifiation of members of the *An. gambiae *complex was performed on individual mosquitoes according to Scott et al. [22].

### SSOP-ELISA for *kdr *detection

**Figure 1:**
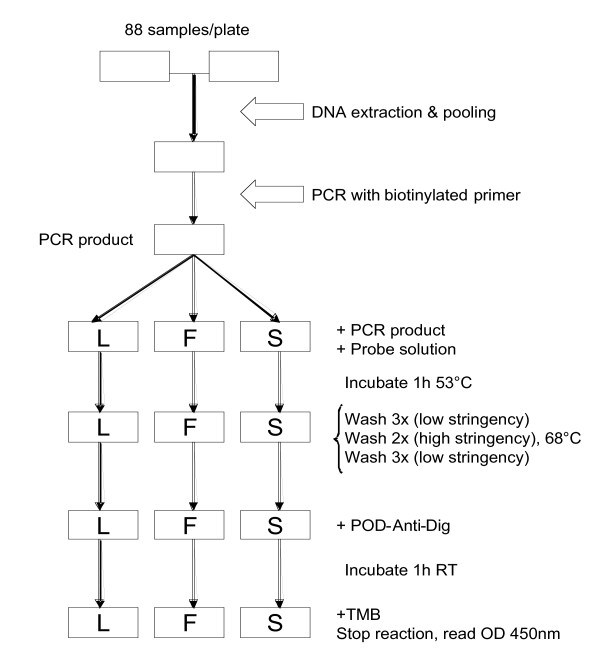
Flow diagram of the SSOP-ELISA procedure for detection of knockdown resistance (*kdr*) in pooled samples using probes corresponding to susceptible (L) and resistant (F, S) genotypes.

The ELISA plates (Maxisorp; Nunc, Roskilde, Denmark) were coated with streptavidin in phosphate buffered saline (PBS) (1 g/mL), covered, and left overnight at 4°C. Prior to use, the plates were washed three times in washing buffer (1 × PBS containing 0.05% Tween 20). The PCR products were diluted 1:1 in water in a 96-well PCR plate, denatured at 95°C for five minutes, and immediately thereafter cooled to 4°C until use. The 3'-end digoxigenin-conjugated SSOPs (MWG Biotech, Riskov, Denmark) (shown in Table 1) were diluted to a 4 nM concentration in tetramethyl ammonium chloride (TMAC; Sigma Aldrich, Dorset, UK) solution (3 M TMAC, 50 mM Tris, pH 8.0, 0.1% sodium dodecyl sulfate, 2 mM EDTA, pH 8.0), heated to 53°C, and 100 μL was then added to each well of the ELISA plate. Two microlitres of the diluted PCR products was subsequently added. Replicate ELISA plates were made to enable simultaneous probing with SSOPs targeting all three *kdr *genotypes. The plates were incubated in a hybridization oven (AH Diagnostics, Aarhus, Denmark) at 53°C on a shaking device for one hour and washed three times in washing buffer. This was followed by two rounds of washing and incubation (14 minutes per round for 104L and 104S probes, 15 minutes per round for the 104F probe) in TMAC solution at 68°C. To remove TMAC, the plates were then washed three times in washing buffer, and peroxidase-conjugated anti-digoxigenin antibody in dilution buffer (1:1,000) (Roche Diagnostics, Mannheim, Germany) was added to each well. After incubation for one hour at room temperature, the plates were washed three times in washing buffer and 100 μL of room temperature TMB substrate (Sigma Aldrich, Dorset, UK) was added to the plates. The reaction was stopped after five minutes by adding 0.5 M H_2_SO_4 _and the optical density (OD) at 450 nm was measured in an ELISA reader. A flow diagram of the methodology is presented in Figure 1.

**Table 1:** Sequence-specific oligonucleotide (SSOP) sequences used for detection of *kdr *single nucleotide polymorphisms (SNPs)

SSOP	SSOP sequence	*kdr *SNP	Reference strain
*104L	GGAAAT**TTA**GTCGTAAGT	wildtype	R70/Dondotha
*104F	GGAAAT**TTT**GTCGTAAGT	West African *kdr*	VKPR
*104S	GGAAAT**TCA**GTCGTAAGT	East African *kdr*	RSP

*position 104 of the *Anopheles gambiae *voltage-gated sodium channel protein sequence, EMBL accession number **Y13592**

### Scoring of ELISA data

For each probe, the non-complementary control strains served as negative controls (e.g. for the 104L probe, wells that contained R70 served as positive controls while wells on the same plate that contained VKPR or RSP served as negative controls). Some between-experiment variation in the OD values of positive and negative controls was apparent, possibly due to marginal differences in the probe binding strength and the washing force during high stringency washes. While this rarely compromised specificity, no fixed threshold could be specified for SNP tests and for each experiment a threshold of positivity was set for each SSOP corresponding to twice the maximum negative control OD value. Samples with OD values exceeding this threshold for each SSOP were considered positive for the corresponding SNP, or combination of SNPs in the case of heterozygotes.

### Evaluation of SSOP-ELISA compared to multiplex PCR

A set of 12 samples, consisting of six individual controls, five mixtures of controls and one field-collected sample were used in a double blind trial to test the consistency of the SSOP-ELISA method in relation to the multiplex PCR methods described by Martinez-Torres et al. [4] and Ranson et al. [6]. The susceptible Dondotha strain of *An. arabiensis *was used in addition to R70 as a wildtype control.

### Sequencing

*Kdr *primers with additional 5' EcoR1 and 3' Not1 site were used to generate PCR products, which were cloned into pAcGP67 (BD biosciences). Plasmids were prepared using MiniPrep spin columns (Omega Biotech). Sequencing was done on an ABI Prism 377 (Perkin-Elmer) using the Big Dye terminator reaction mix (Perkin-Elmer) and ABI Prism proof-reading and translation software.

## Results

### Specificity and sensitivity of SSOP-ELISA

**Figure 2:**
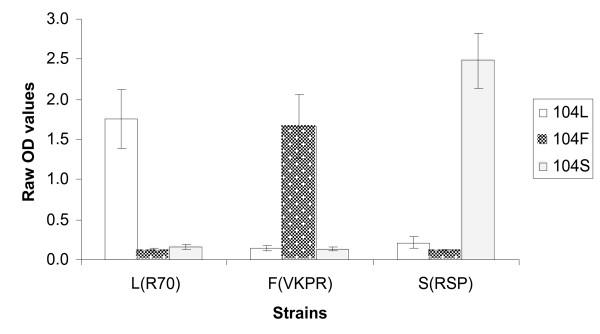
Specificity of SSOP-ELISA to detect control strains of known *kdr *genotype (L, R70; F, VKPR; S, RSP). Mean optical density (OD) and 95% confidence intervals from 10 separate experiments.

**Figure 3:**
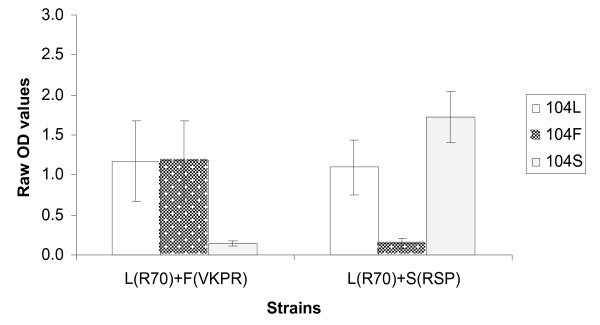
Specificity of SSOP-ELISA to detect *kdr *heterozygotes. Mean optical density (OD) and 95% confidence intervals from 9 separate experiments. Artificial heterozygotes created by combination of DNA from two control strains (L, R70; F, VKPR; S, RSP).

**Figure 4:**
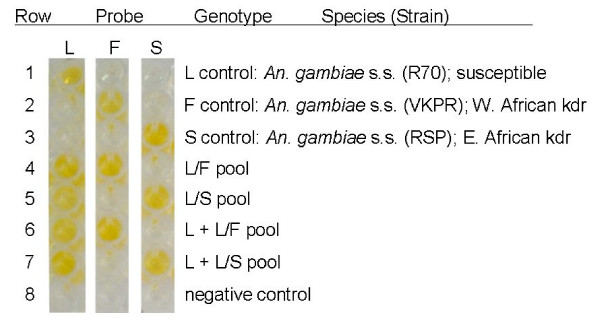
Three columns of an ELISA plate showing visual results obtained for SSOP-ELISA analysis of control strains of *An. gambiae *s.s., individually and in combination.

To evaluate the specificity and sensitivity of the ELISA, the three reference strains of *An. gambiae *s.s., R70, VKPR and RSP known to carry the different *kdr *genotypes, as well as the artificial heterozygote mixtures of these reference strains, were tested. DNA extracted from samples by STE or the Collins method worked equally well, and further evaluation was carried out using DNA extracted by the STE method. Figure 2 shows that the assay correctly identifies the SNPs of the different strains using SSOP for 104L, 104F and 104S for single individuals. The difference in OD value between the threshold of positivity and the positive reactions, ΔOD, was always greater than 1.3. For mixtures of two individuals representing artificial heterozygotes (Figure 3), the OD obtained with *kdr*-specific probes (104F or 104S) was slightly lower than the OD obtained with corresponding probes for single individuals; however, the ΔODs for the different SNPs were at least 0.9. The OD using the wildtype probe (104L) was lower in both mixtures compared to single individuals, with a ΔOD of 0.9 for the L(R70)/F(VKPR) mixture and 0.8 for the L(R70)/S(RSP) mixture; however, the signal strength was sufficient for visual detection. Figure 4 shows the visual results of the SSOP-ELISA test for control strains, individually, in combination as artificial heterozygotes, and with an additional dilution, in three columns of an ELISA plate representing the different SSOP.

In two dilution series with pools containing four or five mosquitoes possessing the resistant and susceptible genotypes, the SSOP-ELISA could reliably detect one resistant individual in a pool containing three other susceptible individuals (1F:3L or 1S:3L), with the ΔOD in relation to the negative control of 0.9 for 104F and 0.7 for 104S. However, when the pool size was increased to five individuals, sensitivity to detect one resistant mosquito in a pool containing four other susceptible individuals (1F:4L or 1S:4L) was reduced, and the ΔOD for the minority genotype was <0.5.

### Evaluation of SSOP-ELISA versus multiplex PCR

Results of the double blind trial are presented in Table 2. All six individual controls were successfully genotyped by multiplex PCR and correctly identified by SSOP-ELISA. For five artificial heterozygotes, multiplex PCR failed to amplify the 137 bp susceptible band in both L/S mixtures, and failed to amplify the 195 bp resistant band in one of two L/F mixtures, while the F/S mixture was successfully genotyped. This was in contrast to the SSOP-ELISA which detected both SNPs in all mixtures with success. The field sample of unknown genotype was identified as 104L by multiplex PCR and SSOP-ELISA.

**Table 2:** Double blind trial to compare results SSOP-ELISA for *kdr *genotyping to results of standard multiplex PCR methods using control strains of known genotype (L, Dondotha/R70; F, VKPR; S, RSP) as individuals or in mixtures.

Test Sample	Genotype results by method		
Genotype(Strain)	SSOP-ELISA	Multiplex PCR	
		
		East African *kdr*^1^	West African *kdr*^2^

L(Dond)+F(VKPR)	L/F	L	L/X
L(Dond)+S(RSP)	L/S	X/S	L
F(VKPR)	F	--	F
field sample	L	L	L
S(RSP)	S	S	--
L(Dond)	L	L	L
F(VKPR)+S(RSP)	F/S	S	F
L(R70)+F(VKPR)	L/F	L	L/F
L(R70)+S(RSP)	L/S	X/S	L
L(R70)	L	L	L
F(VKPR)	F	--	F
S(RSP)	S	S	--

^1^method of Ranson et al. (2000); L = susceptible band present, S = *kdr *band present

^2^method of Martinez-Torres et al. (1998); L = susceptible band present, F = *kdr *band present

-- = band absent (genotype does not correspond to *kdr*-specific detection method)

X = band absent (genotype not detected by corresponding method)

### Analysis of field-collected samples

To ensure detection of heterozygous individuals in field-collected specimens, pools of two individuals only were used representing a total of four alleles. The individuals from positive pools were tested separately for confirmation. All 978 individuals from susceptibility tests and experimental huts in Mabogini tested homozygous for the wildtype susceptible (104L) genotype. However, the SSOP-ELISA detected two out of 642 individuals from Msitu wa Tembo village that were heterozygous for the L104F *kdr *genotype (allele frequency = 0.16%). These L104F *kdr *genotypes were confirmed by DNA sequencing. It was not possible to correlate the *kdr *genotype with insecticide susceptibility status since these mosquitoes had been collected by light trap catch for the purpose of sporozoite rate measurement.

## Discussion

A SSOP-ELISA method is described that allows simple, high-throughput detection of *kdr *SNPs in *An. gambiae *s.l. This method can be established and operated in malaria endemic countries to assist local or national insecticide resistance monitoring programmes. Multiplex PCR methods are often unreliable for detection of heterozygotes [7,16] and other methods have apparent limitations in terms of time and cost. The SSOP-ELISA method has an advantage of using equipment that is readily available in research laboratories in malaria endemic countries. In addition its increased sensitivity can facilitate detection of the *kdr *alleles when still present at low frequency, as demonstrated by detection of two heterozygotes in Tanzania where the *kdr *mutation has not previously been recorded.

While the leucine-phenylalanine *kdr *mutation is widespread in West Africa, it has not, until recently, been found further east of the Central African Republic [12,23]. In Uganda, this mutation was found in combination with the leucine-serine *kdr *mutation in *An. gambiae *s.s. [7]. Interestingly, the presence of the leucine-phenylalanine *kdr *allele in two *An. arabiensis *from northern Tanzania represents the first account of this mutation in East African populations of this sibling species. More widespread monitoring of the distribution of both *kdr *mutations in the *An. gambiae *complex across Africa may therefore reveal less definite geographic restrictions than previously thought. The *kdr *genotype of *An. arabiensis *from Msitu wa Tembo village could not be linked to the resistance phenotype, yet this finding raises potential implications for malaria control. While high frequencies of the same *kdr *mutation in *An. gambiae *s.s. in Côte d'Ivoire have not impacted on the control achieved with insecticide-treated nets [24,25], *kdr *may interact with other genes to eventually have a serious impact on malaria control. The small-scale farming village where *kdr *was detected in the present study is situated nearby the irrigated rice-growing Mabogini area of Tanzania, where survivors of bioassays did not posess the *kdr *genotype [26] suggesting that an additional resistance mechanism, possibly metabolic, may be present in the local vector population.

Present insecticide use in the area where the two *kdr *heterozygotes were collected is negligible, with only small-scale, subsistence agricultural production. However, the river that serves as a focus for vector breeding in this village [27] flows downstream from large-scale agricultural areas where pyrethroids and organochlorines have previously been used (Kulkarni et al. in preparation). Early insecticide use may have selected for resistance in local vector populations, and the mutation may have since existed at low undetectable frequencies. Resistance monitoring using bioassays has not detected significant reductions in insecticide susceptibility in this area (Kulkarni et al. in preparation). However, there is clearly the potential for resistance development in populations where the *kdr *mutation exists at low levels [14] as was demonstrated in western Kenya, where the *kdr *L104S allele pre-dated ITN use and increased significantly in frequency after introduction of ITN [12]. Investigation of local heterogeneity in the frequency of *kdr *in Tanzania and neighbouring countries in relation to history of insecticide use may reveal areas that contributed to early selection of resistance and environmental conditions that might be favouring growth of resistance.

Continued monitoring of vector populations is essential and will benefit from screening for genetic markers of resistance. The occurrence of the typically West African leucine-phenylalanine *kdr *mutation in Tanzania emphasizes the need to test for both *kdr *mutations regardless of geographic location. Use of the cost-effective, high-throughput SSOP-ELISA method to detect *kdr *alleles may contribute to resistance monitoring efforts in many regions of Africa, where little is known on the insecticide resistance status of malaria vector populations.

## Authors' contributions

Manisha Kulkarni organized and coordinated the field work, developed and validated the assay and wrote the first and subsequent drafts of the manuscript.

Michael Alifrangis and Ali Salanti developed the assay and performed the validatory sequencing.

Robert Malima, Eliningaya Kweka, Issa Lyimo and Stephen Magesa performed the field work and mosquito collections.

Johnson Matowo and Justin Peter processed mosquitoes and perfomed SSOP-ELISA assays.

Mark Rowland, Manfred Rau, Frank Mosha and Chris Drakeley conceived the project, oversaw its implementation and contributed to the writing of the manuscript.

All authors have seen and contributed to revisions of the manuscript.
